# Supplementary material for: Stable developmental patterns of gene expression without morphogen gradients
Source: PLoS Comput Biol. 2024 Dec 2;20(12):e1012555. doi: 10.1371/journal.pcbi.1012555 (PMC11661646; doi:10.1371/journal.pcbi.1012555)
Supplement: S1 Text — Containing 5 supporting sections describing additional model details and analysis, 12 supporting figures, and a table of model parameters. (PDF) [file pcbi.1012555.s001.pdf]

SUPPLEMENTARY INFORMATION

Stable developmental patterns of gene expression without morphogen gradients

Maciej Majka[1,2], Nils B. Becker[3,4], Pieter Rein ten Wolde[3], Marcin Zagorski[1], and Thomas R. Sokolowski[3,5,*]

1 Institute of Theoretical Physics and Mark Kac Center for Complex Systems Research, Jagiellonian University, Kraków, Poland
2 Department of Physics, East Carolina University, Greenville, North Carolina, USA
3 AMOLF, Amsterdam, The Netherlands
4 Theoretical Systems Biology, German Cancer Research Center, Heidelberg, Germany
5 Frankfurt Institute for Advanced Studies (FIAS), Frankfurt am Main, Germany

$*$ Corresponding author, e-mail: sokolowski@fias.uni-frankfurt.de

S1.1 Chemical equations describing the gene regulatory network

The biochemical system that we model is governed by the following set of chemical equations:

Monomer production, dimerization, monomer and dimer degradation

$$\mathsf{P}^{\mathsf{A}} \xrightarrow{\beta} \mathsf{A} \qquad \mathsf{A} \underset{k_{\mathrm{off}}^{\mathrm{D}}}{\overset{k_{\mathrm{on}}^{\mathrm{D}}}{\rightleftharpoons}} \mathsf{A}_2 \qquad \mathsf{A} \xrightarrow{\mu_{\mathrm{M}}} \varnothing \qquad \mathsf{A}_2 \xrightarrow{\mu_{\mathrm{D}}} \varnothing$$

Promoter repression and derepression (1st repressor dimer binding)

$$\mathsf{P}^{\mathsf{A}} \underset{k_{\mathrm{s}}^{\mathrm{off}}}{\overset{k_{\mathrm{on}}^{\mathrm{R}}[\mathsf{C}_2]}{\rightleftharpoons}} \mathsf{P}^{\mathsf{A}}_{\mathsf{C}_2} \qquad \mathsf{P}^{\mathsf{A}} \underset{k_{\mathrm{w}}^{\mathrm{off}}}{\overset{k_{\mathrm{on}}^{\mathrm{R}}[\mathsf{B}_2]}{\rightleftharpoons}} \mathsf{P}^{\mathsf{A}}_{\mathsf{B}_2} \qquad \mathsf{P}^{\mathsf{A}} \underset{k_{\mathrm{w}}^{\mathrm{off}}}{\overset{k_{\mathrm{on}}^{\mathrm{R}}[\mathsf{D}_2]}{\rightleftharpoons}} \mathsf{P}^{\mathsf{A}}_{\mathsf{D}_2}$$

Promoter repression and derepression (2nd repressor dimer binding)

$$\mathsf{P}^{\mathsf{A}}_{\mathsf{B}_2} \underset{k_{\mathrm{s}}^{\mathrm{off}}}{\overset{k_{\mathrm{on}}^{\mathrm{R}}[\mathsf{C}_2]}{\rightleftharpoons}} \mathsf{P}^{\mathsf{A}}_{\mathsf{B}_2\mathsf{C}_2} \qquad \mathsf{P}^{\mathsf{A}}_{\mathsf{C}_2} \underset{k_{\mathrm{w}}^{\mathrm{off}}}{\overset{k_{\mathrm{on}}^{\mathrm{R}}[\mathsf{B}_2]}{\rightleftharpoons}} \mathsf{P}^{\mathsf{A}}_{\mathsf{B}_2\mathsf{C}_2}$$

$$\mathsf{P}^{\mathsf{A}}_{\mathsf{B}_2} \underset{k_{\mathrm{w}}^{\mathrm{off}}}{\overset{k_{\mathrm{on}}^{\mathrm{R}}[\mathsf{D}_2]}{\rightleftharpoons}} \mathsf{P}^{\mathsf{A}}_{\mathsf{B}_2\mathsf{D}_2} \qquad \mathsf{P}^{\mathsf{A}}_{\mathsf{D}_2} \underset{k_{\mathrm{w}}^{\mathrm{off}}}{\overset{k_{\mathrm{on}}^{\mathrm{R}}[\mathsf{B}_2]}{\rightleftharpoons}} \mathsf{P}^{\mathsf{A}}_{\mathsf{B}_2\mathsf{D}_2}$$

$$\mathsf{P}^{\mathsf{A}}_{\mathsf{C}_2} \underset{k_{\mathrm{w}}^{\mathrm{off}}}{\overset{k_{\mathrm{on}}^{\mathrm{R}}[\mathsf{D}_2]}{\rightleftharpoons}} \mathsf{P}^{\mathsf{A}}_{\mathsf{C}_2\mathsf{D}_2} \qquad \mathsf{P}^{\mathsf{A}}_{\mathsf{D}_2} \underset{k_{\mathrm{s}}^{\mathrm{off}}}{\overset{k_{\mathrm{on}}^{\mathrm{R}}[\mathsf{C}_2]}{\rightleftharpoons}} \mathsf{P}^{\mathsf{A}}_{\mathsf{C}_2\mathsf{D}_2}$$

Promoter repression and derepression (3rd repressor dimer binding)

$$\mathsf{P}^{\mathsf{A}}_{\mathsf{B}_2\mathsf{D}_2} \underset{k_{\mathrm{s}}^{\mathrm{off}}}{\overset{k_{\mathrm{on}}^{\mathrm{R}}[\mathsf{C}_2]}{\rightleftharpoons}} \mathsf{P}^{\mathsf{A}}_{\mathsf{B}_2\mathsf{C}_2\mathsf{D}_2} \qquad \mathsf{P}^{\mathsf{A}}_{\mathsf{B}_2\mathsf{C}_2} \underset{k_{\mathrm{w}}^{\mathrm{off}}}{\overset{k_{\mathrm{on}}^{\mathrm{R}}[\mathsf{D}_2]}{\rightleftharpoons}} \mathsf{P}^{\mathsf{A}}_{\mathsf{B}_2\mathsf{C}_2\mathsf{D}_2} \qquad \mathsf{P}^{\mathsf{A}}_{\mathsf{C}_2\mathsf{D}_2} \underset{k_{\mathrm{w}}^{\mathrm{off}}}{\overset{k_{\mathrm{on}}^{\mathrm{R}}[\mathsf{B}_2]}{\rightleftharpoons}} \mathsf{P}^{\mathsf{A}}_{\mathsf{B}_2\mathsf{C}_2\mathsf{D}_2}$$

Note that here, for brevity, we only show the complete set of reactions for the promoter of gene A, and its products (A monomers and dimers A_2). An analogous set of equations holds for the promoters of genes B, C and D, with P^A replaced by P^B, P^C or P^D, respectively, all occurrences of the corresponding dimers (B_2, C_2 or D_2) replaced by A_2 and corresponding monomers A replaced by B, C, or D, respectively, and the unbinding rates adjusted to the respective strength of mutual interactions.

In addition to the shown set of equations, we provide a graph-like summary of the reaction network in Fig. L.

S1.2 Analysis of perturbation experiments for assessing pattern restoring forces

Perturbation experiments on patterns initially relaxed into their long-persisting intact state were carried out by expanding expression domains at the expense of the respective antagonistic gene's domains and simulating the subsequent dynamics of the system, as described in the main text and Methods, Sec. 4. The stochastic time trajectories of such perturbation experiments that were repeated many times were then analysed as follows:

In order to quantify the spatial properties of a given domain G we used its center of mass, z_{G}, where G stands for one of the five domains $\{A_{\mathrm{a}}, B, C, D, A_{\mathrm{p}}\}$, with A_{a} and A_{p} marking the anterior and posterior parts of A. Advantageously, z_{G} is a robust measure, as it avoids ambiguity associated with determining domain boundaries in the presence of gene expression noise. Figure A shows, for $\kappa = \kappa_{\mathrm{opt}}$, time traces of z_{G} for the two types of perturbations, averaged over 10 independent samples in each case. For both perturbations the average centers of copy number relax back to their original positions on a timescale $\sim 10\,h$. This demonstrates that for optimal repression strength ratio an effective restoring force counteracts deviations from the five-stripe pattern for varied λ_{AC}.

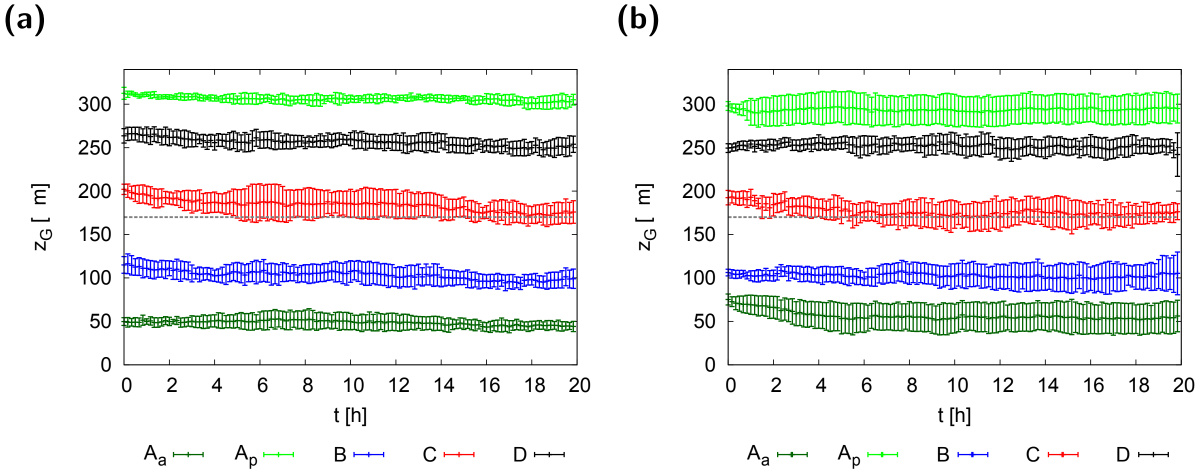

Figure A. Perturbed trajectories are restored to their origin at optimal NN repression strength. Shown are averaged time traces of the copy number center-of-masses z_{G} for the five domains of the stable pattern ($\mathrm{A_a}$ = anterior A domain, $\mathrm{A_p}$ = posterior A domain) at $\kappa = \kappa_{\mathrm{opt}}$ for two different perturbations: (a) "C expansion", i.e. prolongation of the central C domain by $\Delta = 8$ nuclei into the posterior, and (b) "A expansion", i.e. prolongation of the anterior A domain by $\Delta = 8$ nuclei towards the center of the embryo. The gray-dashed line marks the center of the system. In both cases we observe a restoration of the metastable state on a timescale $\lesssim 10$ h.

S1.3 Estimation of phase space diffusion coefficient from overdamped Langevin dynamics

Let $f(X, Y, t)$ be a twice differentiable real function depending on a two-dimensional diffusion-drift processes $\vec{X} = (X, Y)$ and time t (explicitly). In the overdamped Langevin limit, i.e. assuming that the displacements of the random walker are governed only by forces that stem from an underlying force field and by Gaussian noise, and that its accelerations and inertia are negligible, we can describe this random processes via

$$d\vec{X} = \vec{v}dt + \sigma d\vec{W} \tag{S1}$$

where $\vec{W}$ is a (two-dimensional) Wiener processes and $\vec{v} = (v_{\mathrm{X}}, v_{\mathrm{Y}})$ a (local) drift velocity resulting from the potential forces.

We then can calculate the differential of f with ITŌ's Lemma (as a generalization of Taylor expansion) as follows:

$$\begin{aligned} df(X,Y,t) = &\sigma \frac{\partial f}{\partial X} dW_{\mathrm{X}} + \sigma \frac{\partial f}{\partial Y} dW_{\mathrm{Y}} \\ &+ \left[\frac{\partial f}{\partial t} + v_{\mathrm{X}} \frac{\partial f}{\partial X} + v_{\mathrm{Y}} \frac{\partial f}{\partial Y} + \frac{\sigma^2}{2} \frac{\partial^2 f}{\partial X^2} + \frac{\sigma^2}{2} \frac{\partial^2 f}{\partial Y^2} + \zeta \sigma^2 \frac{\partial^2 f}{\partial X \partial Y} \right] dt \end{aligned} \tag{S2}$$

Here ζ measures the correlation between X and Y.

In order to apply this general formula to the specific diffusion-drift problem for the phase space coordinates $(\lambda_{\mathrm{AC}}, \lambda_{\mathrm{BD}})$ defined in the main text Eq. (2), we assign for brevity $\lambda_{\mathrm{x}} = \lambda_{\mathrm{AC}}$ and $\lambda_{\mathrm{y}} = \lambda_{\mathrm{BD}}$, and then we set $X = \lambda_{\mathrm{x}}, Y = \lambda_{\mathrm{y}}$ and $f(X, Y, t) = f(\lambda_{\mathrm{x}}, \lambda_{\mathrm{y}}) = (\lambda_{\mathrm{x}} - \lambda_{\mathrm{x}_0})^2 + (\lambda_{\mathrm{y}} - \lambda_{\mathrm{y}_0})^2 \equiv \Delta\lambda^2$ (the squared displacement function).

Itō's Lemma now reads (note that the time and mixed derivatives vanish):

$$\begin{aligned} d(\Delta\lambda^2) &= d\left[(\Delta\lambda_{\mathrm{x}})^2 + (\Delta\lambda_{\mathrm{y}})^2\right] = d\left[(\lambda_{\mathrm{x}} - \lambda_{\mathrm{x}_0})^2 + (\lambda_{\mathrm{y}} - \lambda_{\mathrm{y}_0})^2\right] \\ &\simeq 2(\lambda_{\mathrm{x}} - \lambda_{\mathrm{x}_0})(v_{\lambda_{\mathrm{x}}} dt + \sigma dW_{\mathrm{x}}) + 2(\lambda_{\mathrm{y}} - \lambda_{\mathrm{y}_0})(v_{\lambda_{\mathrm{y}}} dt + \sigma dW_{\mathrm{y}}) \\ &\quad + \frac{\sigma^2}{2} 2dt + \frac{\sigma^2}{2} 2dt \end{aligned} \tag{S3}$$

To relate the above formula to the displacements sampled in our simulations with a fixed acquisition time interval Δt we shall integrate the infinitesimal contributions over this interval. At the same time we take the ensemble average to account for the averaging of independent samples, which causes the Gaussian terms σdW_{x} and σdW_{y} to vanish. We further assume that, to a good approximation, the drift velocities and diffusion coefficients are constant over the time interval Δt and diffusion isotropic in λ_{x} and λ_{y} direction, i.e. $D_{\lambda_{\mathrm{x}}} = D_{\lambda_{\mathrm{y}}} = D_{\lambda}(\vec{\lambda})$.

Finally, using $\sigma = \sqrt{2D_\lambda}$, we obtain:

$$\begin{aligned}
\left\langle \Delta\lambda^2 \right\rangle &= \left\langle \int_{\Delta \mathrm{t}} d(\Delta\lambda^2) \right\rangle \\
&= \left\langle \int_0^{\Delta t} 2 \underbrace{\left[\lambda_{\mathrm{x}}(t) - \lambda_{\mathrm{x}}(0)\right]}_{\simeq \langle \mathrm{v}_{\lambda_{\mathrm{x}}} \rangle \mathrm{t}} \underbrace{v_{\lambda_{\mathrm{x}}}}_{\simeq \langle \mathrm{v}_{\lambda_{\mathrm{x}}} \rangle} dt \right\rangle + \left\langle \int_0^{\Delta t} 2 \underbrace{\left[\lambda_{\mathrm{y}}(t) - \lambda_{\mathrm{y}}(0)\right]}_{\simeq \langle \mathrm{v}_{\lambda_{\mathrm{y}}} \rangle \mathrm{t}} \underbrace{v_{\lambda_{\mathrm{y}}}}_{\simeq \langle \mathrm{v}_{\lambda_{\mathrm{y}}} \rangle} dt \right\rangle \\
&\quad + \left\langle \int_0^{\Delta t} 4 D_\lambda dt \right\rangle + \int_{\Delta \mathrm{t}} \underbrace{\langle 2\Delta\lambda_{\mathrm{x}} \sigma dW_{\mathrm{x}} \rangle}_{0} + \int_{\Delta \mathrm{t}} \underbrace{\langle 2\Delta\lambda_{\mathrm{y}} \sigma dW_{\mathrm{y}} \rangle}_{0} \\
&\simeq \left\langle \langle v_{\lambda_{\mathrm{x}}} \rangle^2 \int_0^{\Delta t} 2t dt \right\rangle + \left\langle \langle v_{\lambda_{\mathrm{y}}} \rangle^2 \int_0^{\Delta t} 2t dt \right\rangle + 4 \langle D_\lambda \rangle \Delta t \\
&\simeq \langle v_{\lambda_{\mathrm{x}}} \Delta t \rangle^2 + \langle v_{\lambda_{\mathrm{y}}} \Delta t \rangle^2 + 4 \langle D_\lambda \rangle \Delta t \\
&= \langle \Delta\lambda_{\mathrm{x}} \rangle^2 + \langle \Delta\lambda_{\mathrm{y}} \rangle^2 + 4 \langle D_\lambda \rangle \Delta t
\end{aligned} \tag{S4}$$

The final result shows that, knowing the average displacements $\langle \Delta\lambda_{\mathrm{x}} \rangle$ and $\langle \Delta\lambda_{\mathrm{x}} \rangle$ and average squared displacements $\left\langle \Delta\lambda^2 \right\rangle$ at $\vec{\lambda}$, we can compute the average diffusion coefficient $\langle D_\lambda \rangle (\vec{\lambda})$ via:

$$\langle D_\lambda \rangle (\vec{\lambda}) = \frac{1}{4\Delta t} \left[\left\langle \Delta\lambda^2 \right\rangle (\vec{\lambda}) - \left(\langle \Delta\lambda_{\mathrm{x}} \rangle^2 (\vec{\lambda}) + \langle \Delta\lambda_{\mathrm{y}} \rangle^2 (\vec{\lambda}) \right) \right] = \frac{1}{4\Delta t} \mathcal{V}_{\langle \lambda \rangle}(\vec{\lambda}) \tag{S5}$$

The bracket term containing the first moments corrects the mean squared displacement for the contributions coming from the deterministic drift and tends to zero as the process becomes purely diffusive.

The results of this analysis applied to our simulated systems are shown in Fig. B, where we plot the diffusion coefficients and the resulting expected diffusion times to the edge of the basin of initial patterns as a function of our main parameter, the repression strength ratio κ. Interestingly, we find that the diffusion constant remains almost constant for large κ, corresponding to weak nearest-neighbor repression, see Fig. B, panel (a). In this regime, the estimated time of diffusion to the edge of the stable basin, $\tau_{0.2}$ is about 12 hours and comparable to the recorded average stability times ($\sim$ 20 hours, panels (b) and (c)). When κ is reduced, meaning that the nearest-neighbor repression is increased, we observe an increase of the diffusion constant and correspondingly a reduction of $\tau_{0.2}$ to values below 10 hours. Since in this regime the pattern stability increases dramatically, our analysis corroborates the finding that the stability increase is not due to a slow-down of the pattern boundary dynamics, but due to the emergence and deepening of a metastable basin that generates restoring forces to pattern perturbations.

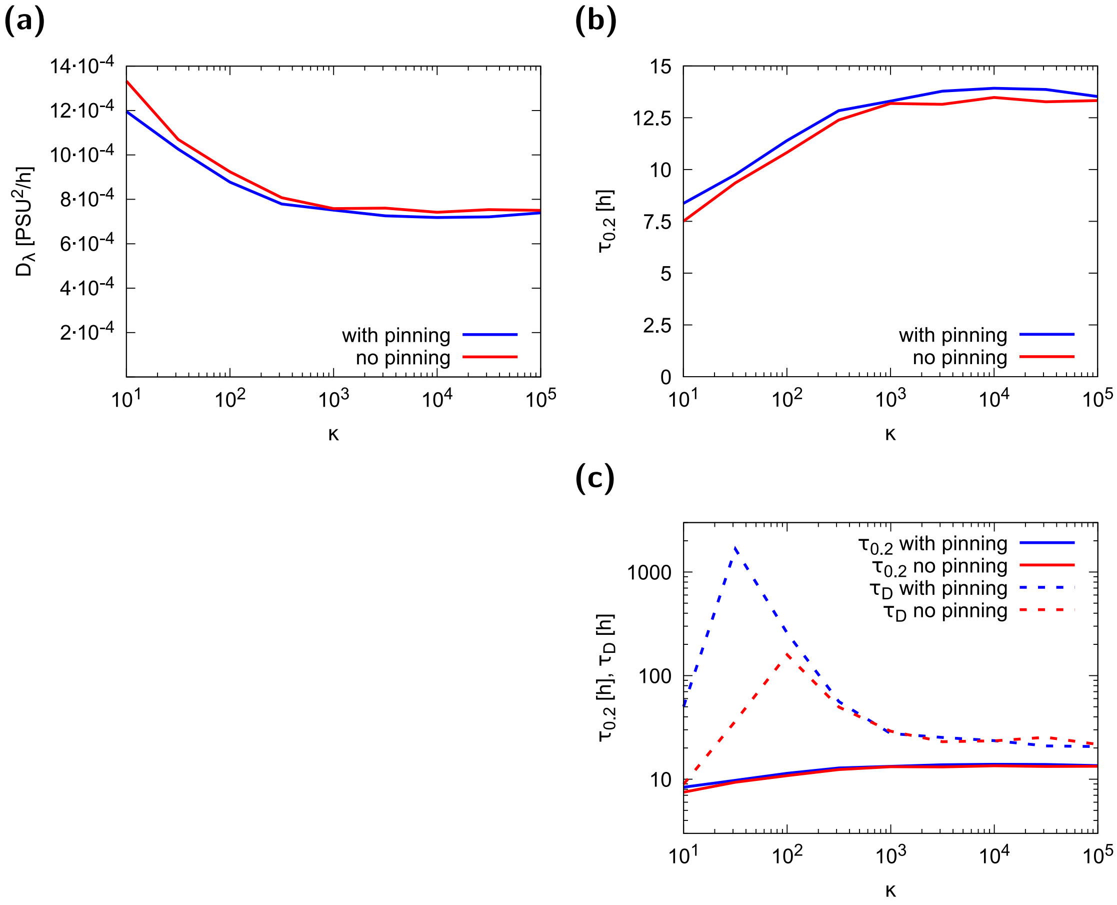

Figure B. Average phase space diffusion coefficients as a function of κ. ((a)) The diffusion coefficient of phase space trajectories in the $(\lambda_{\mathrm{x}}, \lambda_{\mathrm{y}}) = (\lambda_{\mathrm{AC}}, \lambda_{\mathrm{BD}})$ space are obtained from the overdamped Langevin analysis, see section S1.3. The diffusion coefficients are averaged over the phase space region $R_{\mathrm{P}} = [0.3, 0.4]^2$, which is part of the diffusive plateau, for different repression strength ratios κ. The PSU stands for the phase space units. ((b)) The resulting approximate diffusion times from the phase space region of the five-stripe relaxed patterns, towards the edge of the diffusive plateau as a function of κ, assuming a distance of 0.2 PSU for the initial phase space distance to the edge. The edge of diffusive plateau is defined as a region of $(\lambda_{\mathrm{AC}}, \lambda_{\mathrm{BD}})$ from which the states are quickly absorbed into the regions with one the domains lost.

S1.4 Supplementary velocity field figures

Figures C, D, E and F show phase space velocity fields and trajectories starting from perturbed initial conditions for three different projections of the reaction coordinates, for the following cases:

- for the repression strength ratio $\kappa \simeq 31.6$ that optimizes pattern stability with pinning, see Fig. C,
- for weaker NN repression strength $\kappa = 1000$ with pinning, see Fig. D,
- for the optimal repression strength ratio $\kappa = 100$ that optimizes pattern stability without pinning, see Fig. E,
- for weaker NN repression strength $\kappa = 1000$ without pinning, see Fig. F.

S1.5 Example pattern destruction processes

Figures G, H and I summarize typical pattern destruction processes traced in biased simulated time (within the NS-FFS scheme) as the reaction coordinate $\lambda = \lambda_{\rm AC} + \lambda_{\rm BD}$ increases, for the system with pinning and at optimal repression strength ratio $\kappa \simeq 30$ (maximally stable regime).

In all figures, panel (a) shows snapshots of the circumference-averaged expression patterns, initially consisting of four expression domains, some of which are destroyed as λ increases. Panels (b) show the corresponding increase of λ. Panels (c) illustrate how the four gene expression domain sizes (defined as the number of positions at which the expression level of the respective gene is above a predefined threshold of 3 copies) change as biased simulation time and λ increase–initially starting from approximately equal domain sizes, some domains are eliminated while their counterpart domains increase in size and ultimately can span the whole system.

More specifically, Fig. G shows an example destruction process in which the expression domain of gene C (red) is destroyed first. Notice that once the C domain is eliminated, this appears to enable subsequent expansion of the D domain (black) at the expense of the opposing B domain (blue).

Fig. H shows an example in which first the B domain (blue) is destroyed by the counterpart D domain (black). This is accompanied by a steady shrinkage of the C domain (red) by the A domain (green); however, the A domain does not manage to destroy the C domain completely by the end of the simulated time interval.

In contrast, Fig. I shows a case in which two genes manage to destroy their opposing counterparts' expression domains by that time point: First the A domain grows at the expense of the C domain, while growth of B at the expense of D follows afterwards with reduced speed. Notice that the early asymmetry between B and D is transiently reduced before B starts to push out D ultimately completely, highlighting a (partial) pattern restoration event.

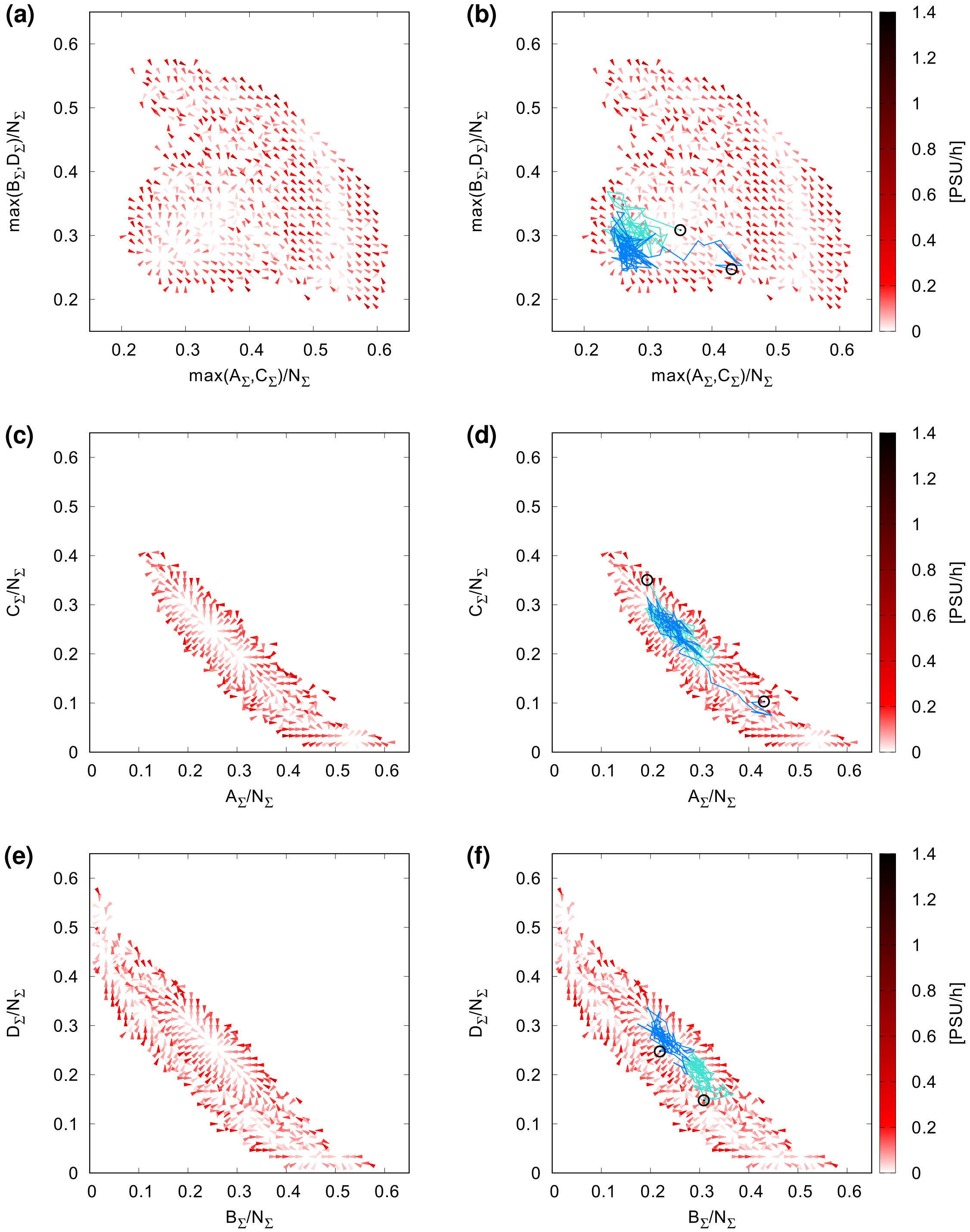

Figure C. Average phase space velocities for the maximally stable system ($\kappa \simeq 31.6$) with pinning. Left plots (a, c, e) show local average phase space velocities, right plots (b, d, f) additionally show example trajectories for the two types of perturbations considered in the restoration experiments (blue = pert. from boundary, turquoise = pert. from center). Starting points are marked by black bullets.

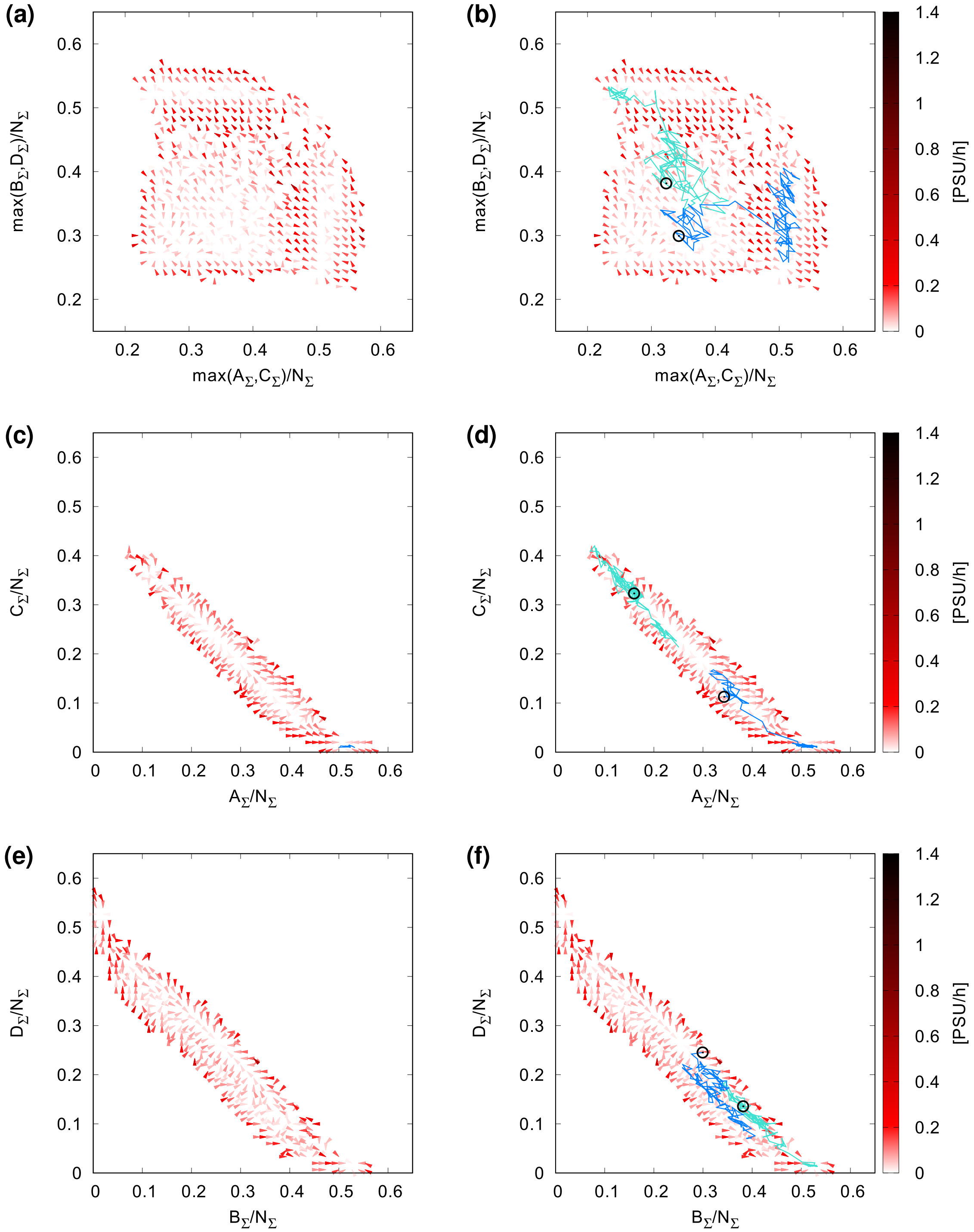

Figure D. Average phase space velocities for weaker NN interaction ($\kappa = 1000$) in the system with pinning. Left plots (a, c, e) show local average phase space velocities, right plots (b, d, f) additionally show example trajectories for the two types of perturbations considered in the restoration experiments (blue = pert. from boundary, turquoise = pert. from center). Starting points are marked by black bullets.

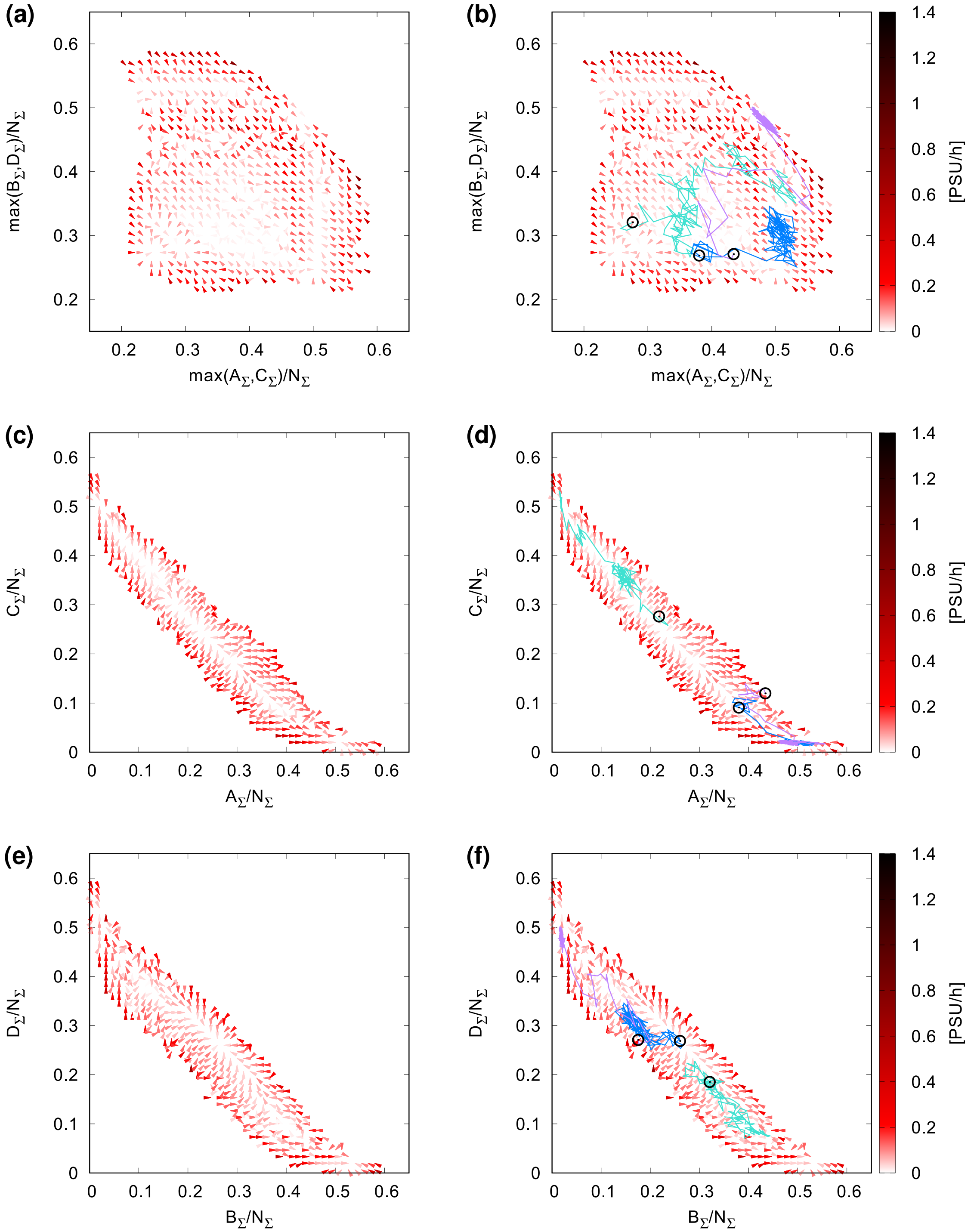

Figure E. Average phase space velocities for the maximally stable system ($\kappa = 100$) without pinning. Left plots (a, c, e) show local average phase space velocities, right plots (b, d, f) additionally show example trajectories for the two types of perturbations considered in the restoration experiments (blue = pert. from boundary, turquoise = pert. from center; purple = pert. from center resulting in complete pattern destruction). Starting points are marked by black bullets.

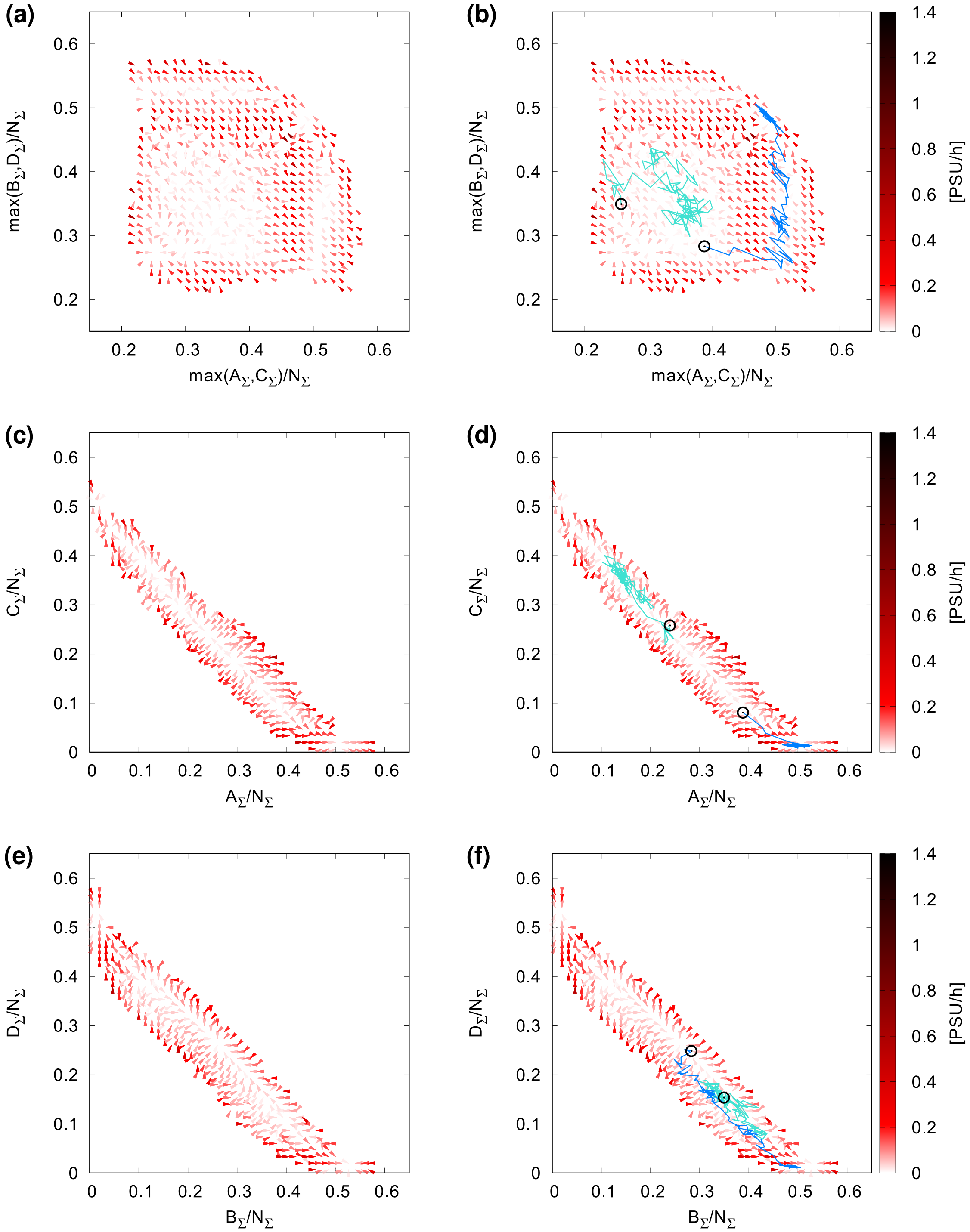

Figure F. Average phase space velocities for weaker NN interaction ($\kappa = 1000$) in the system without pinning. Left plots (a, c, e) show local average phase space velocities, right plots (b, d, f) additionally show example trajectories for the two types of perturbations considered in the restoration experiments (blue = pert. from boundary, turquoise = pert. from center). Starting points are marked by black bullets.

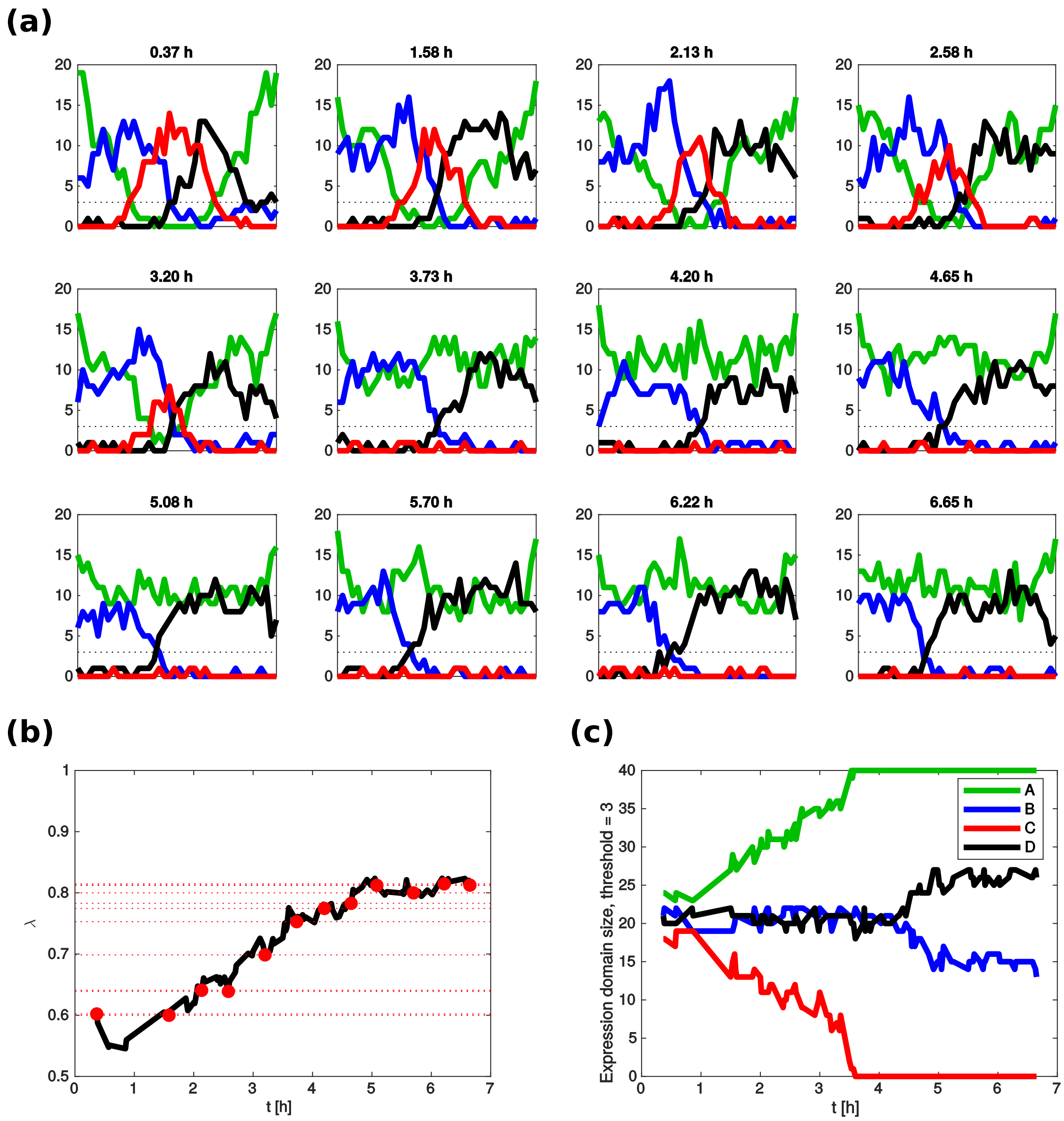

Figure G. Example destruction event: Destruction of the C domain at optimal NN repression strength ($\kappa = \kappa_{\rm opt} \simeq 30$) in the system with pinning. Panel (a) shows snapshots of gene expression patterns (averaged along the circumference) at the indicated times of the biased simulation trajectory. Panel (b) shows the progress of the reaction coordinate $\lambda = \lambda_{\rm AC} + \lambda_{\rm BD}$ along the simulated time trajectory; red bullets correspond to the snapshots in (a) in the same temporal order, red dotted lines show the corresponding λ levels. Panel (c) shows the corresponding evolution of the expression domain sizes of the four individual genes, here defined by the number of positions at which the (circumference-averaged) expression level is above 3 copies (dotted lines in (a) panels). The C domain (red) starts to disappear from 2.13 h onwards and is completely destroyed by the A domain (green) by 3.73 h. Notice the posterior movement of the boundary between the B and D domains that appear to precede A destruction in this case. Later the B-D boundary moves back towards the anterior again.

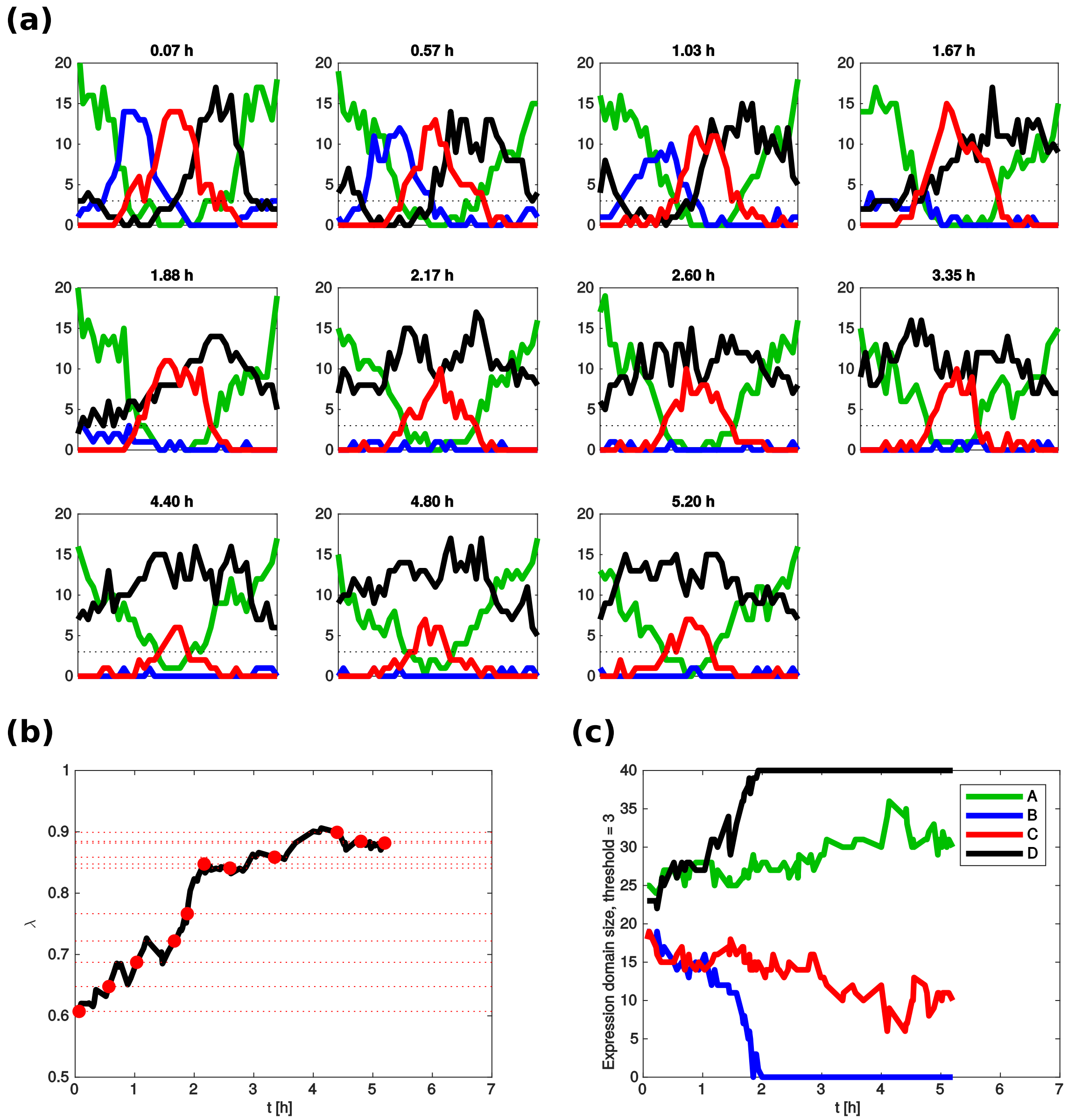

Figure H. Example destruction event: Destruction of the B domain at optimal NN repression strength ($\kappa = \kappa_{\rm opt} \simeq 30$) in the system with pinning. Panel (a) shows snapshots of gene expression patterns (averaged along the circumference) at the indicated times of the biased simulation trajectory. Panel (b) shows the progress of the reaction coordinate $\lambda = \lambda_{\rm AC} + \lambda_{\rm BD}$ along the simulated time trajectory; red bullets correspond to the snapshots in (a) in the same temporal order, red dotted lines show the corresponding λ levels. Panel (c) shows the corresponding evolution of the expression domain sizes of the four individual genes, here defined by the number of positions at which the (circumference-averaged) expression level is above 3 copies (dotted lines in (a) panels). The B domain (blue) starts to be suppressed by the D domain (black) already from 1.03 h onwards and is completely destroyed by 2.17 h. Notice that the destruction of the B domain appears to be accompanied by a compression of the C (red) domain by the posterior A (green) domain at 0.57 h.

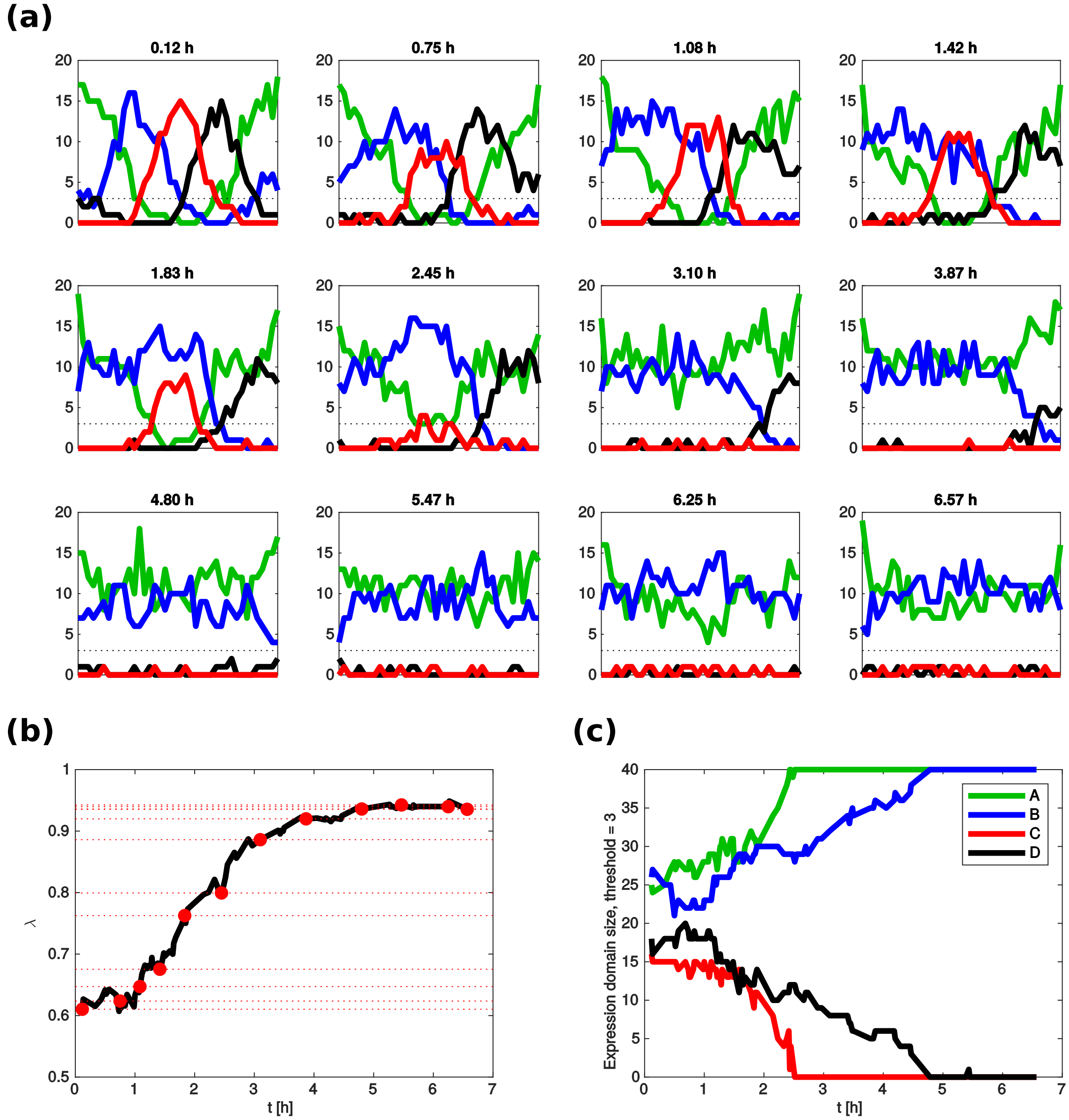

Figure I. Example destruction event: Destruction of both the C and D domains at optimal NN repression strength ($\kappa = \kappa_{\rm opt} \simeq 30$) in the system with pinning. Panel (a) shows snapshots of gene expression patterns (averaged along the circumference) at the indicated times of the biased simulation trajectory. Panel (b) shows the progress of the reaction coordinate $\lambda = \lambda_{\rm AC} + \lambda_{\rm BD}$ along the simulated time trajectory; red bullets correspond to the snapshots in (a) in the same temporal order, red dotted lines show the corresponding λ levels. Panel (c) shows the corresponding evolution of the expression domain sizes of the four individual genes, here defined by the number of positions at which the (circumference-averaged) expression level is above 3 copies (dotted lines in (a) panels). Notice that in this example the C (red) domain is under pressure already at 0.75 h, but then rebounds again at 1.08 h, demonstrating the pattern's restoring capability. In parallel, the interface between B (blue) and D (black) moves posteriorly, which initiates the destruction of the C domain at 1.83 h, completed by 3.10 h. Subsequently, the B domain also expels the D domain across the posterior system boundary, enabled by the fact that D is not pinned there (in contrast to A).

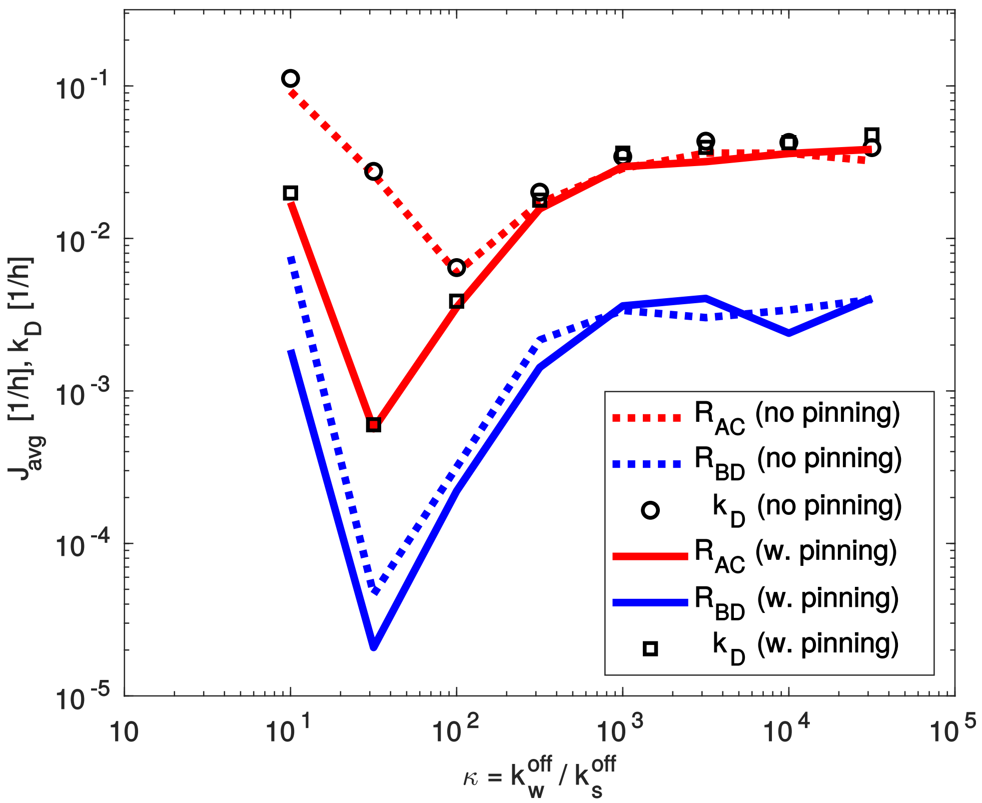

Figure J. Pinning affects destruction pathways We plot here the average probability fluxes from the region of stable patterns $R_{\rm S}$ into the different remote basins identified in the $(\lambda_{\rm AC}, \lambda_{\rm BD})$ phase space as a function of the repression strength ratio κ for the systems with and without pinning. Here the flux is defined as the average increase per time of the total probability in the basin. Basin boundaries and flux quantities are described in detail in Methods, Sec. 4. Shown are the flux into the basin $R^{\dagger}_{\rm AC}$, corresponding to destruction of either the A or C domain (red lines), the flux into the basin $R^{\dagger}_{\rm BD}$, in which either the B or D domain breaks down (blue lines), and the total outflux from $R_{\rm S}$, which equals the pattern destruction rate $k_{\rm D}$ (black bullets). Solid lines and triangles show the data for the system with pinning, dashed lines and circles the values for the system without pinning. Clearly, in both with and without pinning and for all κ considered here, $R^{\dagger}_{\rm AC}$ is the dominant fraction of the flux, reflecting that the dominant pathway to destruction is the one that starts with the disappearance of either the A or C domain. Pinning of A expression at the system boundaries leads to a pronounced reduction of the flux through this pathway for $\kappa = 10 - 100$.

S1.6 Pinning alters the proportion of destruction pathways

Both with and without pinning of A expression at the system boundaries, pattern stability is maximal at an optimal strength of NN repression. Stability times, however, are significantly higher in the system with pinning. In order to understand whether this is simply due to the fact that pinning prohibits destruction of the A domains or due to other pinning-induced effects, we compared the different pathways to destruction by computing probability fluxes through distinct reaction pathways (see Methods, Sec. 4 for details). The different reaction pathways are defined by the order in which gap gene domains are destroyed. In our system there are two major pathways: the A-C destruction pathway (either the A or the C domain vanishes first) and the B-D destruction pathway (either the B or D domain vanishes first). The phase space histograms in Figure 3 of the main text demonstrate that simultaneous destruction of two domains, corresponding to trajectories that progress diagonally in $(\lambda_{\mathrm{AC}}, \lambda_{\mathrm{BD}})$ space, is highly improbable. We find that, while in general the A-C destruction pathway prevails, the fact that the A-destruction pathway is dominant for $\kappa \leq 100$ in the system without pinning accounts for the strong enhancement of pattern stability due to pinning.

In Figure J we plot for different repression strength ratios κ the magnitude of average fluxes from the region of intact patterns R_{S} in the $(\lambda_{\mathrm{AC}}, \lambda_{\mathrm{BD}})$ space into the respective neighboring regions that correspond to states in which one expression domain vanished. The figure reveals that for all κ the flux through the A-C destruction pathway is approximately ten times higher than the flux through the B-D pathway, for systems both with and without pinning. The figure also shows that pinning indeed reduces the flux through the dominant, i.e. A-C, pathway, most significantly for $\kappa \simeq 10 - 100$, i.e. around the optimal value κ_{opt}. This gives rise to the pronounced stability enhancement. The simultaneous reduction of the flux through the B-D pathway is not relevant for overall stability.

We analysed further the detailed composition of fluxes through the dominant (A-C) pathway by computing the average flux into the regions of destroyed states in $([A]_{\mathrm{tot}}/N_{\mathrm{tot}}, [C]_{\mathrm{tot}}/N_{\mathrm{tot}})$ space, see Fig. K. As expected, in the systems with pinning the entire flux through the dominant pathway goes into the C-destroyed state. Interestingly, this is also the case for the weakly coupled systems without pinning. Here the flux into the A-destroyed state is clearly dominant over the flux into the C-destroyed state for strong NN interaction. This explains why pinning, which prohibits exit through the B-destruction pathway, increases stability in the $\kappa \lesssim 100$ regime. While the flux through the C-destruction pathway is minimal at $\kappa = 31.6$ with or without pinning, in the system without pinning the accessibility of A destruction shifts the minimum of the combined flux through both pathways towards $\kappa = 100$, see Fig. J.

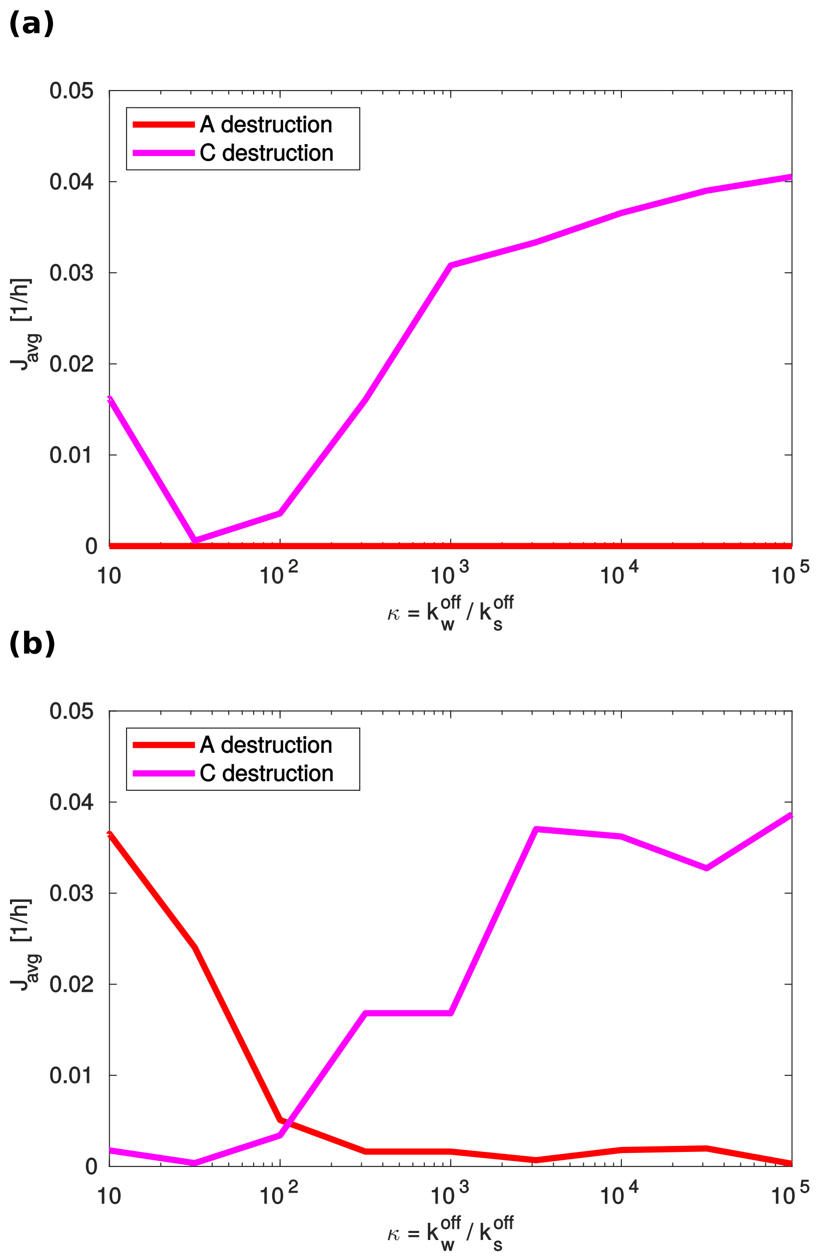

Figure K. Pinning shifts the destruction flux balance in the dominant (A-C) destruction pathway The figure shows the contributions of the A-destruction and C-destruction pathways to the outflux from R_S as a function of the repression strength ratio κ for the systems with (a) and without (b) pinning. See Methods, Sec. 4 for the definitions of basin boundaries and details of flux calculation. Without pinning and for strong NN repression, the preferred pathway to destruction is the one in which the A domains are destroyed first, while for weaker coupling (large κ) destruction begins via annihilation of the C domain. Interestingly, in both cases the flux through the C-destruction pathway is minimal at $\kappa \simeq 31.6$. However, in the system without pinning this value falls into the regime in which the flux through the A-pathway markedly increases. Pinning forbids destruction via the A pathway and thus dramatically reduces the overall destruction flux for low κ in the system with pinning, giving rise to the additional enhancement of optimal stability at $\kappa \simeq 31.6$.

Quantity	Symbol	Value	Unit
Geometry			
Nuclear radius	r_{N}	2.5	$\mu\mathrm{m}$
Nuclear volume	V_{N}	65.4	$\mu\mathrm{m}^3$
No. of nuclei in axial direction	N_{z}	40	
- resulting system length	L	340	$\mu\mathrm{m}$
No. of nuclei in circumferential direction	N_{ϕ}	8	
Production / degradation			
Protein production rate	β	0.20	s^{-1}
Monomer degradation rate	μ_{M}	0.05	s^{-1}
Dimer degradation rate	μ_{D}	0.005	s^{-1}
- resulting effective degr. rate	μ_{eff}	0.0095	s^{-1}
Binding / unbinding			
Intranuclear diffusion const.	D_{N}	3.2	$\mu\mathrm{m}^2/s$
Repressor target site radius	σ_{R}	0.5	$\mu\mathrm{m}$
- resulting (diff. ltd.) repressor on-rate	$k_{\mathrm{on}}^{\mathrm{R}}$	20.1	$\mu\mathrm{m}^3/s$
Standard (strong) repressor off-rate	$k_{\mathrm{s}}^{\mathrm{off}}$	0.06	s^{-1}
Weak repressor off-rate	$k_{\mathrm{w}}^{\mathrm{off}}$	varied	$\geq k_{\mathrm{off}}^{\mathrm{R,s}}$
Monomer protein radius	σ_{M}	0.05	$\mu\mathrm{m}$
- resulting (diff. ltd.) dimerization forward rate	$k_{\mathrm{on}}^{\mathrm{D}}$	4.0	$\mu\mathrm{m}^3/s$
Dimerization backward rate	$k_{\mathrm{off}}^{\mathrm{D}}$	0.062	$\mu\mathrm{m}^3/s$
Internuclear diffusion			
Standard internuclear diffusion const.	D_{P}	1.0	$\mu\mathrm{m}^2/s$
Internuclear lattice distance	l	8.5	$\mu\mathrm{m}$

Table A. The standard parameters of the simulated model of four mutually repressing genes prearranged in the "alternating cushions" pattern on a cylindrical lattice of expressing nuclei.

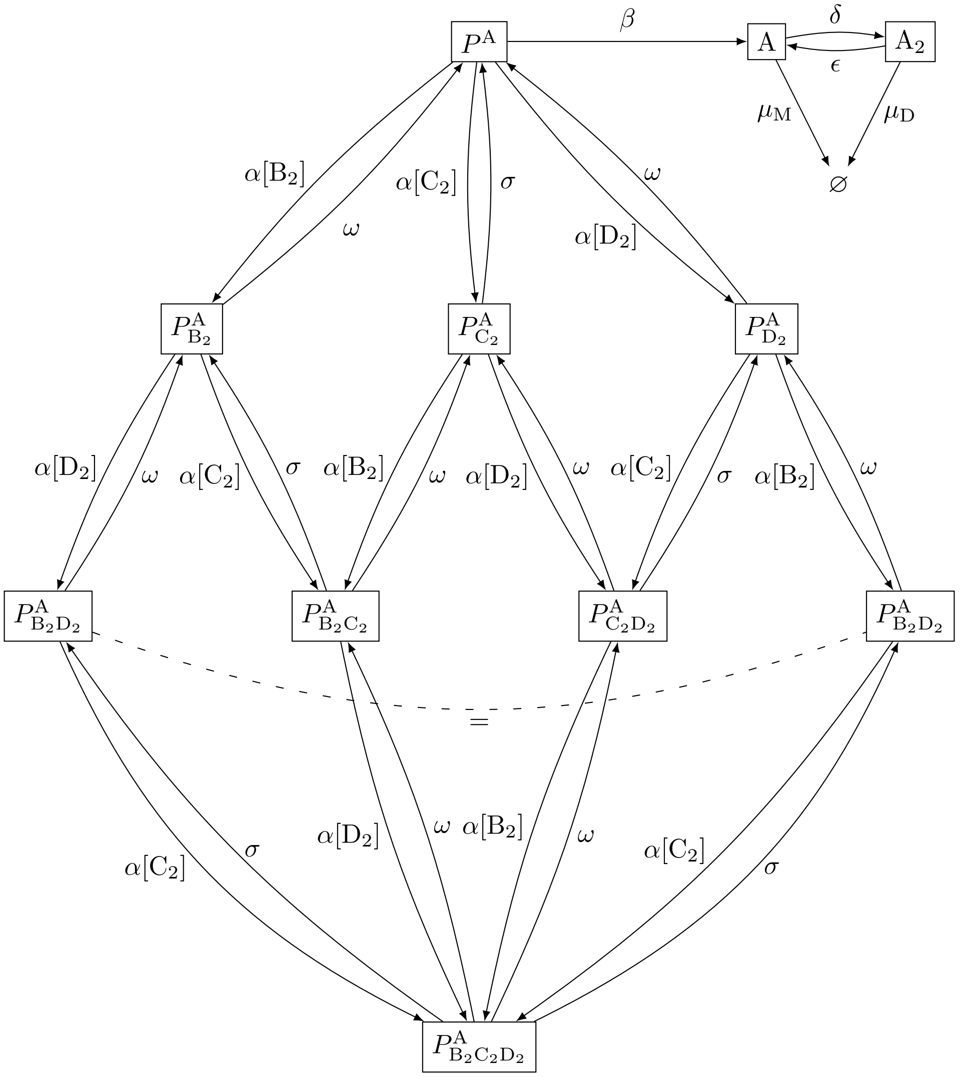

Figure L. Reaction network. This schematic shows the set of reactions that affect production and degradation of a single gap gene species A. The strong repressor of A is denoted by B, the weak interaction partners by C and D. For each species, X denotes the monomer, X_2 the dimer. For easy readability here we abbreviate: $\alpha \equiv k_{\mathrm{on}}^{\mathrm{R}}$ = diffusion limited repressor binding rate; $\sigma \equiv k_{\mathrm{s}}^{\mathrm{off}}$ = next-nearest neighbor / strong repressor unbinding rate; $\omega \equiv k_{\mathrm{w}}^{\mathrm{off}}$ = nearest-neighbor / weak repressor unbinding rate; $\delta \equiv k_{\mathrm{on}}^{\mathrm{D}}$ = dimerization forward rate; $\epsilon \equiv k_{\mathrm{off}}^{\mathrm{D}}$ = dimerization backward rate. The schematic shows the reactions for the promoter of species A and its protein products, which we denote by $(\mathrm{A}||\mathrm{C},\mathrm{B},\mathrm{D})$, but holds similarly for all other combinations of regulated and regulating species, $(\mathrm{B}||\mathrm{D},\mathrm{A},\mathrm{C})$, $(\mathrm{C}||\mathrm{A},\mathrm{B},\mathrm{D})$ and $(\mathrm{D}||\mathrm{B},\mathrm{A},\mathrm{C})$; the order of the regulating species is not strictly alphabetical because the strongly repressing species is indicated first.

List of Figures

A **Perturbed trajectories are restored to their origin at optimal NN repression strength.** Shown are averaged time traces of the copy number center-of-masses z_{G} for the five domains of the stable pattern ($\mathrm{A_a}$ = anterior A domain, $\mathrm{A_p}$ = posterior A domain) at $\kappa = \kappa_{\mathrm{opt}}$ for two different perturbations: (a) "C expansion", i.e. prolongation of the central C domain by $\Delta = 8$ nuclei into the posterior, and (b) "A expansion", i.e. prolongation of the anterior A domain by $\Delta = 8$ nuclei towards the center of the embryo. The gray-dashed line marks the center of the system. In both cases we observe a restoration of the metastable state on a timescale $\lesssim 10$ h. 2

B **Average phase space diffusion coefficients as a function of κ.** ((a)) The diffusion coefficient of phase space trajectories in the $(\lambda_{\mathrm{x}}, \lambda_{\mathrm{y}}) = (\lambda_{\mathrm{AC}}, \lambda_{\mathrm{BD}})$ space are obtained from the overdamped Langevin analysis, see section S1.3. The diffusion coefficients are averaged over the phase space region $R_{\mathrm{P}} = [0.3, 0.4]^2$, which is part of the diffusive plateau, for different repression strength ratios κ. The PSU stands for the phase space units. ((b)) The resulting approximate diffusion times from the phase space region of the five-stripe relaxed patterns, towards the edge of the diffusive plateau as a function of κ, assuming a distance of 0.2 PSU for the initial phase space distance to the edge. The edge of diffusive plateau is defined as a region of $(\lambda_{\mathrm{AC}}, \lambda_{\mathrm{BD}})$ from which the states are quickly absorbed into the regions with one the domains lost. 5

C **Average phase space velocities for the maximally stable system ($\kappa \simeq 31.6$) with pinning.** Left plots (a, c, e) show local average phase space velocities, right plots (b, d, f) additionally show example trajectories for the two types of perturbations considered in the restoration experiments (blue = pert. from boundary, turquoise = pert. from center). Starting points are marked by black bullets. 7

D **Average phase space velocities for weaker NN interaction ($\kappa = 1000$) in the system with pinning.** Left plots (a, c, e) show local average phase space velocities, right plots (b, d, f) additionally show example trajectories for the two types of perturbations considered in the restoration experiments (blue = pert. from boundary, turquoise = pert. from center). Starting points are marked by black bullets. 8

E **Average phase space velocities for the maximally stable system ($\kappa = 100$) without pinning.** Left plots (a, c, e) show local average phase space velocities, right plots (b, d, f) additionally show example trajectories for the two types of perturbations considered in the restoration experiments (blue = pert. from boundary, turquoise = pert. from center; purple = pert. from center resulting in complete pattern destruction). Starting points are marked by black bullets. 9

F **Average phase space velocities for weaker NN interaction ($\kappa = 1000$) in the system without pinning.** Left plots (a, c, e) show local average phase space velocities, right plots (b, d, f) additionally show example trajectories for the two types of perturbations considered in the restoration experiments (blue = pert. from boundary, turquoise = pert. from center). Starting points are marked by black bullets. 10

G **Example destruction event: Destruction of the C domain at optimal NN repression strength ($\kappa = \kappa_{\rm opt} \simeq 30$) in the system with pinning.** Panel (a) shows snapshots of gene expression patterns (averaged along the circumference) at the indicated times of the biased simulation trajectory. Panel (b) shows the progress of the reaction coordinate $\lambda = \lambda_{\rm AC} + \lambda_{\rm BD}$ along the simulated time trajectory; red bullets correspond to the snapshots in (a) in the same temporal order, red dotted lines show the corresponding λ levels. Panel (c) shows the corresponding evolution of the expression domain sizes of the four individual genes, here defined by the number of positions at which the (circumference-averaged) expression level is above 3 copies (dotted lines in (a) panels). The C domain (red) starts to disappear from 2.13 h onwards and is completely destroyed by the A domain (green) by 3.73 h. Notice the posterior movement of the boundary between the B and D domains that appear to precede A destruction in this case. Later the B-D boundary moves back towards the anterior again. 11

H **Example destruction event: Destruction of the B domain at optimal NN repression strength ($\kappa = \kappa_{\rm opt} \simeq 30$) in the system with pinning.** Panel (a) shows snapshots of gene expression patterns (averaged along the circumference) at the indicated times of the biased simulation trajectory. Panel (b) shows the progress of the reaction coordinate $\lambda = \lambda_{\rm AC} + \lambda_{\rm BD}$ along the simulated time trajectory; red bullets correspond to the snapshots in (a) in the same temporal order, red dotted lines show the corresponding λ levels. Panel (c) shows the corresponding evolution of the expression domain sizes of the four individual genes, here defined by the number of positions at which the (circumference-averaged) expression level is above 3 copies (dotted lines in (a) panels). The B domain (blue) starts to be suppressed by the D domain (black) already from 1.03 h onwards and is completely destroyed by 2.17 h. Notice that the destruction of the B domain appears to be accompanied by a compression of the C (red) domain by the posterior A (green) domain at 0.57 h. 12

I **Example destruction event: Destruction of both the C and D domains at optimal NN repression strength ($\kappa = \kappa_{\rm opt} \simeq 30$) in the system with pinning.** Panel (a) shows snapshots of gene expression patterns (averaged along the circumference) at the indicated times of the biased simulation trajectory. Panel (b) shows the progress of the reaction coordinate $\lambda = \lambda_{\rm AC} + \lambda_{\rm BD}$ along the simulated time trajectory; red bullets correspond to the snapshots in (a) in the same temporal order, red dotted lines show the corresponding λ levels. Panel (c) shows the corresponding evolution of the expression domain sizes of the four individual genes, here defined by the number of positions at which the (circumference-averaged) expression level is above 3 copies (dotted lines in (a) panels). Notice that in this example the C (red) domain is under pressure already at 0.75 h, but then rebounds again at 1.08 h, demonstrating the pattern's restoring capability. In parallel, the interface between B (blue) and D (black) moves posteriorly, which initiates the destruction of the C domain at 1.83 h, completed by 3.10 h. Subsequently, the B domain also expels the D domain across the posterior system boundary, enabled by the fact that D is not pinned there (in contrast to A). 13

J **Pinning affects destruction pathways** We plot here the average probability fluxes from the region of stable patterns R_{S} into the different remote basins identified in the $(\lambda_{\mathrm{AC}}, \lambda_{\mathrm{BD}})$ phase space as a function of the repression strength ratio κ for the systems with and without pinning. Here the flux is defined as the average increase per time of the total probability in the basin. Basin boundaries and flux quantities are described in detail in Methods, Sec. 4. Shown are the flux into the basin $R^{\dagger}_{\mathrm{AC}}$, corresponding to destruction of either the A or C domain (red lines), the flux into the basin $R^{\dagger}_{\mathrm{BD}}$, in which either the B or D domain breaks down (blue lines), and the total outflux from R_{S}, which equals the pattern destruction rate k_{D} (black bullets). Solid lines and triangles show the data for the system with pinning, dashed lines and circles the values for the system without pinning. Clearly, in both with and without pinning and for all κ considered here, $R^{\dagger}_{\mathrm{AC}}$ is the dominant fraction of the flux, reflecting that the dominant pathway to destruction is the one that starts with the disappearance of either the A or C domain. Pinning of A expression at the system boundaries leads to a pronounced reduction of the flux through this pathway for $\kappa = 10 - 100$. 14

K **Pinning shifts the destruction flux balance in the dominant (A-C) destruction pathway** The figure shows the contributions of the A-destruction and C-destruction pathways to the outflux from R_{S} as a function of the repression strength ratio κ for the systems with (a) and without (b) pinning. See Methods, Sec. 4 for the definitions of basin boundaries and details of flux calculation. Without pinning and for strong NN repression, the preferred pathway to destruction is the one in which the A domains are destroyed first, while for weaker coupling (large κ) destruction begins via annihilation of the C domain. Interestingly, in both cases the flux through the C-destruction pathway is minimal at $\kappa \simeq 31.6$. However, in the system without pinning this value falls into the regime in which the flux through the A-pathway markedly increases. Pinning forbids destruction via the A pathway and thus dramatically reduces the overall destruction flux for low κ in the system with pinning, giving rise to the additional enhancement of optimal stability at $\kappa \simeq 31.6$. 16

L **Reaction network.** This schematic shows the set of reactions that affect production and degradation of a single gap gene species A. The strong repressor of A is denoted by B, the weak interaction partners by C and D. For each species, X denotes the monomer, X_2 the dimer. For easy readability here we abbreviate: $\alpha \equiv k^{\mathrm{R}}_{\mathrm{on}}$ = diffusion limited repressor binding rate; $\sigma \equiv k^{\mathrm{off}}_{\mathrm{s}}$ = next-nearest neighbor / strong repressor unbinding rate; $\omega \equiv k^{\mathrm{off}}_{\mathrm{w}}$ = nearest-neighbor / weak repressor unbinding rate; $\delta \equiv k^{\mathrm{D}}_{\mathrm{on}}$ = dimerization forward rate; $\epsilon \equiv k^{\mathrm{D}}_{\mathrm{off}}$ = dimerization backward rate. The schematic shows the reactions for the promoter of species A and its protein products, which we denote by $(\mathrm{A}||\mathrm{C},\mathrm{B},\mathrm{D})$, but holds similarly for all other combinations of regulated and regulating species, $(\mathrm{B}||\mathrm{D},\mathrm{A},\mathrm{C})$, $(\mathrm{C}||\mathrm{A},\mathrm{B},\mathrm{D})$ and $(\mathrm{D}||\mathrm{B},\mathrm{A},\mathrm{C})$; the order of the regulating species is not strictly alphabetical because the strongly repressing species is indicated first. 18

List of Tables

A The standard parameters of the simulated model of four mutually repressing genes prearranged in the "alternating cushions" pattern on a cylindrical lattice of expressing nuclei. 17
